# Histomorphological and ultrastructural cadmium-induced kidney injuries and precancerous lesions in rats and screening for biomarkers

**DOI:** 10.1042/BSR20212516

**Published:** 2022-06-09

**Authors:** Xichen Wan, Zelong Xing, Jin Ouyang, Hui Liu, Chengquan Cheng, Ting Luo, Shiqun Yu, Li Meihua, Shaoxin Huang

**Affiliations:** 1 Department of Neurosurgery, the First Affiliated Hospital of Nanchang University, Nanchang, Jiangxi, 330000, P.R. China; 2 Department of Social Science and Public Health, Medical school, Jiujiang University, Jiujiang, 332000, P.R. China; 3 Jiangxi Provincial Key Laboratory of Preventive Medicine, Nanchang University, Nanchang 330006, P.R. China; 4 School of Public Health, Qingdao University, 16 Ningde Road, Qingdao, Shandong, 266021, P.R. China

**Keywords:** biomarkers, Cadmium, Chemical carcinogenesis, Renal toxicity

## Abstract

Long-term exposure to cadmium (Cd) can severely damage the kidney, where orally absorbed Cd accumulates. However, the molecular mechanisms of Cd-induced kidney damage, especially the early biomarkers of Cd-induced renal carcinogenesis, are unclear. In the present study, we established a rat kidney injury model by intragastric administration of Cd to evaluate the morphological and biochemical aspects of kidney injury. We randomly divided Sprague-Dawley rats into control, low Cd (3 mg/kg), and high Cd (6 mg/kg) groups and measured biochemical indices associated with renal toxicity after 2, 4, and 8 weeks of treatment. The Cd-exposed mice had significantly higher Cd concentrations in blood and renal tissues as well as blood urea nitrogen (BUN), β2-microglobulin (β2-MG), urinary protein excretion, and tumor necrosis factor-α (TNF-α) levels. Furthermore, histopathological and transmission electron microscopy (TEM) observations revealed structural disruption of renal tubules and glomeruli after 8 weeks of exposure to the high Cd regimen. Besides, microarray technology experiments showed that Cd increased the expression of genes related to the chemical carcinogenesis pathway in kidney tissue. Finally, combining the protein–protein interaction (PPI) network of the Cd carcinogenesis pathway genes with the microarray and Comparative Toxicogenomics Database (CTD) results revealed two overlapping genes, CYP1B1 and UGT2B. Therefore, the combined molecular and bioinformatics experiments’ results suggest that CYP1B1 and UGT2B are biomarkers of Cd-induced kidney injury with precancerous lesions.

## Introduction

The heavy metal cadmium (Cd) is a hazardous and toxic industrial and environmental pollutant [1]. Humans can be exposed to Cd through contaminated food, drinking water, air, and soil [2,3]. Cd harms several target organs in humans, including the brain, lung, liver, bones [4,5], and especially the kidney [6], as Cd-induced nephrotoxicity involves apoptosis, necrosis, and atrophy of renal tubular epithelial cells [7]. In addition, the kidneys absorb approximately 50% of the total body Cd after long-term Cd exposure [8]. Thus, long-term Cd exposure can even lead to kidney cancer in humans [9]. However, the molecular mechanisms of Cd-induced nephrotoxicity, especially the molecular mechanisms involving precancerous lesions with Cd as a chemical carcinogen, remain unclear, hindering its clinical treatment [10].

Renal accumulation of Cd reduces the glomerular filtration rate (GFR), which may eventually lead to renal failure and damage the proximal tubules of the kidney [5]. Several models allow researchers to assess foreign body-mediated nephrotoxicity [2,11,12]. Our study used rats as an alternative exogenous nephrotoxicity model. In contrast with the intervention methods of some previous animal models [2,12–14], we used intragastric administration, using experimental doses and intervention times based on previous studies [15–17] to obtain a more stable animal model and facilitate the assessment of molecular mechanisms. Similar to previous findings, indicators such as GFR, urine volume, and blood urea nitrogen (BUN) and serum creatinine (SCR) levels reflect renal function and can thus help assess nephrotoxicity [6]. In the early-disease stages, biomarkers such as β2-microglobulin (β2-MG) can be detected in the urine [18,19]. β2-MG is a low-molecular-weight protein with low plasma levels [15]. Under normal conditions, it is filtered by the glomerulus and then efficiently reabsorbed by the proximal tubule [20]. Thus, β2-MG has been widely used as an early indicator of Cd-induced renal dysfunction in humans [21,22]. Besides, previous studies have suggested that tumor necrosis factor-α (TNF-α) plays a role in nephrotoxicity [23,24]. Therefore, the present study selects BUN, β2-MG, TNF-α, and urinary protein to reflect the characteristics of Cd-induced nephrotoxicity in rat model.

In addition, we performed transmission electron microscopy (TEM), which is rare in similar studies, and documented the effect of Cd on the ultrastructure of the kidney.

Cd is a chemical carcinogen, however, the mechanism of renal carcinogenesis (especially precancerous lesions) induced after Cd-induced kidney injury is unclear. Cd accumulates in the renal cortex and is a well-documented nephrotoxin [6]. Cellular and molecular mechanisms implicated in Cd carcinogenicity include proto-oncogenes activation, tumor-suppressor genes inactivation, cell adhesion disruption, and DNA repair inhibition [25].

In the present study, we established a Cd-induced kidney injury rat model by intragastric administration and documented the molecular and morphological changes. The microarray experiment indicated that Cd enriched chemical carcinogenesis pathway genes in kidney tissue in this model even before morphological kidney tissue carcinogenesis indicators appeared. We also studied and compared the chemical carcinogenesis pathways of Cd using the Comparative Toxicogenomics Database (CTD) to screen for early Cd-induced renal chemical carcinogenesis biomarkers. Some molecules associated with chemical carcinogenesis that cause kidney damage by Cd may serve as early biomarkers of the Cd carcinogenic pathway. Screening for precancerous lesions are molecules on the chemical carcinogenic pathway, which will facilitate early screening and targeted therapeutic targets for Cd carcinogenesis.

## Methods

### Animal treatment and experimental design

We purchased Sprague-Dawley rats from SJA Experimental Animal Co. (Hunan Province, China) (*n*=54, 27 males and 27 females). They were acclimatized for three days before the experiments started. We recorded the rats’ weights before the experiments, then gave them *ad libitum* access to basic chow and ultrapure water. Relative humidity was 50–60% and room temperature was 23–25°C. The experimental procedures followed the International Guide for the Care and Use of Laboratory Animals, and the Jiujiang Institute approved the animal protocols.

All animals were randomly divided into three groups of 18 rats each, as described below. Cadmium chloride (CdCl_2_) was purchased from Shanghai Maclean’s Biochemistry Ltd (Shanghai, China).

- Control group (*n*=18). Each rat received a daily intragastric injection of 1 ml of sodium chloride solution.
- Low Cd group (*n*=18). Each rat received a daily intragastric injection of 1 ml of 3 mg/kg CdCl_2_ solution.
- High Cd group (*n*=18). Each rat received a daily intragastric injection of 1 ml of 6 mg/kg of CdCl_2_ solution.

We collected samples after 2, 4, and 8 weeks of treatment. The rats received adequate daily water supply. During the experiment, we recorded the rats’ water consumption and their body weight daily. The experimental and control groups had a similar weekly water consumption (*P*>0.05).

### Sample collection

One day before the sacrifice, we housed all the rats in separate metabolic cages and collected urine for 24 h. After recording body weight, urine volume, and urine pH, we immediately stored all urine samples in a −80°C refrigerator. We anesthetized the rats by intraperitoneal injection of 10% chloral hydrate (0.3 ml/100 g). Next, we collected blood from the abdominal aorta and centrifuged it at 1500 rpm for 10 min. We then immediately froze the serum samples and stored them in a −80°C refrigerator until analysis. We then removed the kidneys and weighed them. We fixed a small portion of the right kidney in 10% neutral buffered formalin for histopathological studies, froze the left kidney in liquid nitrogen, and stored it in a −80°C refrigerator before Cd quantification. After the kidney was removed, the rats were killed by anesthetic overdose. The animal experiments in the present paper were carried out in the Preventive Medicine Laboratory of Medical College of Jiujiang University in China. The ethics approval number is JJ.No20180601S0540130 [019].

### Cd in the blood and kidneys

We quantified Cd in the serum and renal tissue using an atomic absorption spectrometer (SpectrAA-240FS; Varian, Inc, California, U.S.A.).

### Urinalysis and serum biochemical examinations

We quantified β2-MG, 24 h urinary protein excretion, BUN, and TNF-α levels in thawed urine and serum samples using kits according to the manufacturer’s instructions. We purchased all the reagents used from Nanjing Jiancheng Bioengineering (Nanjing, China).

### Histopathology

The right kidneys of rats were fixed in 10% formalin for at least 24 h, dehydrated using standard procedures, and embedded in paraffin. We cut sections (approximately 4 µm thick), stained them with hematoxylin and eosin (H&E), and examined them under a light microscope. We photographed the specimens using a Nikon Eclipse E200-LED (Tokyo, Japan).

### Western blotting

To compare protein expression in the Cd and control groups, we ground the kidney tissue with protein lysate buffer (TPEB) containing a protease inhibitor (Transgen, Beijing, China) with a glass homogenizer. Next, we determined the extracted proteins concentration using a Qubit™ Fluorocytometer (Invitrogen, U.S.A.) by loading equal amounts of protein onto each well of the Nu PAGE Bis-Tris Gel (Invitrogen, U.S.A.). After adding the protein samples into the loading buffer (Transgen, Beijing, China), we separated them by SDS-PAGE at 80 V for 30 min, then 120 V for 60 min. We then transferred them electrophoretically to polyvinylidene fluoride membranes at 400 mV for 1 h. Next, we blocked the transferred membranes with 10% skim milk and incubated them at 4°C overnight with primary antibody (UDP-glucuronyltransferase 2B (UGT2B), Abcam, ab113433, dilution at 1:200) (Cytochrome P450 (CYP) 1B1, Abcam, ab185954, dilution at 1:100), and (β-Actin, Abcam, ab197277, dilution at 1:1000). After each step, we washed the membranes three-times. Then, we incubated the membranes at room temperature for 2 h with goat antirabbit IgG (Transgen, dilution at 1:1000) for UGT2B and CYP1B1, and goat antimouse IgG (Transgen, dilution at 1:1000) for β-Actin. Finally, we analyzed the blots and quantified the proteins using Image lab software using Bio-Rad Molecular image ChemiDocTM XRS (Bio-Rad, California, U.S.A.). All experiments were performed in triplicate.

### TEM

After 8 weeks of treatment, we fixed the fresh kidney tissues of the high dose group and control group rats overnight at 4°C in 2.5% glutaraldehyde (0.1 mol/l phosphate buffer, pH 7.4). We then fixed them at 4°C with 1% samarium tetroxide in the same buffer for 1 h. Next, we cut ultrathin sections on a Leica EMUC7 ultra-microslicing machine and stained them with lead citrate and uranyl acetate. We observed the changes in renal glomeruli and renal tubules using a Japanese Hitachi HT7700 transmission electron microscope.

### Microarray

We extracted total RNA from rat kidney tissue using Trizol reagent (Invitrogen, Carlsbad, California, U.S.A.) according to the manufacturer’s instructions. We then synthesized double-stranded complementary DNA (dsDNA) using an RT kit (Macherey, Germany). Next, we purified the dsDNA products using a NucleoSpin Extract II Kit (Macherey, Germany) and eluted them with 30 µl elution buffer.

We labeled and hybridized dsDNA using a CapitalBioTechnology (Beijing, China) rat array v1prime8x60K. The CapitalBio Technology rat LncRNA array v1 has four identical arrays (8 × 60K format) for each glass slide, each array containing about 30254 rat mRNA probes. After hybridization and washing, we scanned the processed slides with GeneSpring software V13.0 (Agilent Technologies, Inc.) and transformed the data using a Log_2_. We defined ‘modulated genes’ as those with a fold-change >2 or <−2 and a false discovery rate (Benjamini and Hochberg’s method) corrected *P*-value smaller than 0.05.

### KEGG pathway

We analyzed the EGG pathways of genes expressed differentially in the Cd and control groups using David (https://EGG.fanciful.gov/Summary). We selected the top ten shared fanciful pathways with the smallest *P*-values.

### Construction and analysis of the protein–protein interaction network

We analyzed the protein–protein interaction (PPI) network of differentially expressed genes enriched in chemical carcinogenesis pathways after a Cd-induced renal injury using STRING (http://string-db.org, an online database that can predict and generate a PPI network) and Cityscape (an open-source software used to analyze PPI networks and search for hub genes). We analyzed the degree, tweeness, and closeness for each gene in the network and visualized it using Cityscape 3.7.2. The CTD (http://ctdbase.org/) is a web-based tool providing information on the interactions between chemicals and genetic products and their links to diseases. We analyzed the related genes in the Cd-induced chemical carcinogen pathway using the CTD and constructed and analyzed a PPI network.

### RNA extraction and quantitative reverse transcription polymerase chain reaction

We extracted total RNA from the cells using TRIzol reagent (Life Technologies, Grand Island, NY, U.S.A.) according to the manufacturer’s instructions. We then stored the extracted RNA in a −80°C refrigerator until use. Next, we performed qPCR with a StepOne™ real-time PCR system (Applied Biosystems; Thermo Fisher Scientific, Inc.). We obtained FAST SYBR®Green Master Mix from Applied Biological Systems and used Primer Premier 5 to design specific primers. The primers of the CYP1B1 gene were: forward (5′-3′) GCT CAG CCA CAA CGA GGA GTT C and reverse (5′-3′) CTG GTA AAG AGG ATG AGC. The primers of the UGT2B gene were: forward (5′-3′) CCC TTG CCC AGA TTC CAC AA and reverse (5′-3′) AGG ATG TCA TTC TGC GGG AG. Using the ΔΔCt method and β-actin as an internal control, we compared the relative mRNA levels of CYP1B1 and UGT2B genes in samples. The results were expressed as the mean ± standard deviation of three independent experiments.

### Statistical analysis

Results were expressed as the mean ± standard deviation. We compared the means of the three groups by a one-way analysis of variance (ANOVA) followed by Tukey–Kramer multiple comparison tests. The difference between the groups was considered statistically significant for *P*-values<0.05. GraphPad Prism 6 was used to draw the figures.

## Results

### Construction of a Cd nephrotoxicity model

**Figure 1:**
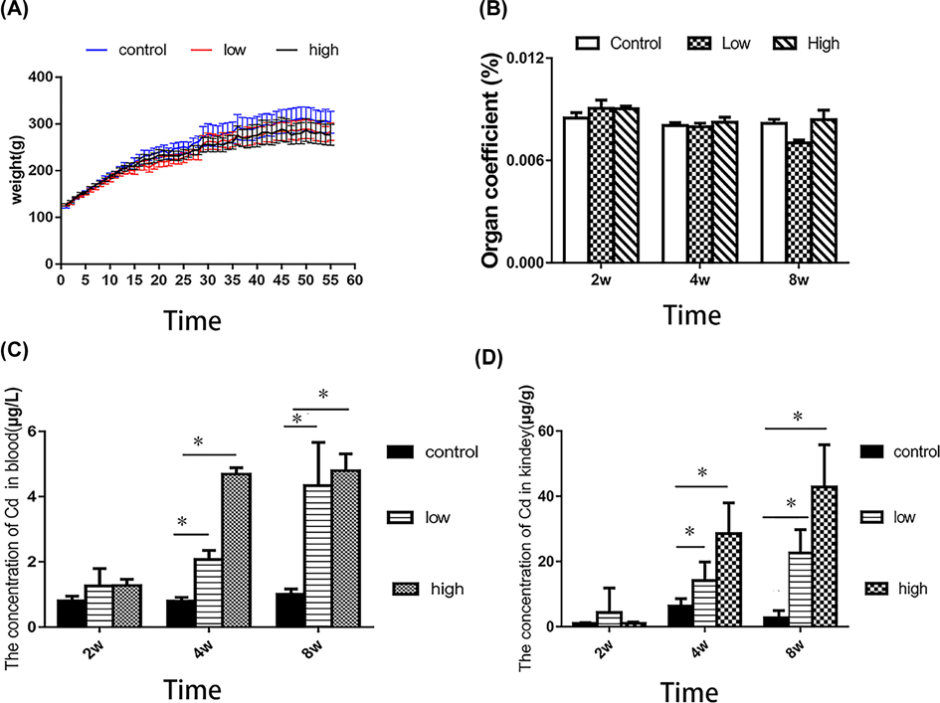
Effects of Cd on body weight, organ coefficient, and Cd concentration in the blood and kidneys (**A**) Effects of Cd exposure on the body weight of rats. (**B**) Effects of Cd exposure on renal organ coefficient in rats. (**C**) Effect of Cd exposure on blood Cd concentration. (**D**) Effect of Cd exposure on kidney Cd concentration. * *P*<0.05 compared with the control group.

During the experiment, the rats were weighed every day. The control and Cd groups had similar body weights and kidney organ coefficients (Figure 1A,B). Cd gavage considerably increased Cd levels in the blood and renal tissue (Figure 1C,D). After 4 weeks of gavage, the low Cd and high Cd groups had blood Cd levels, respectively, 1.97 (95% CI: 1.69–2.47, *P*<0.05) and 4.65 (95% CI: 4.29–4.87, *P*<0.05) times higher than the control group. After 8 weeks, the low Cd and high Cd groups had blood Cd levels, respectively, 4.19 (95% CI: 3.89–4.57, *P*<0.05) and 4.89 (95% CI: 4.77–5.96, *P*<0.05) times higher than the control group (Figure 1C).

In the kidney, 4 weeks of gavage increased the Cd levels by 1.87-fold (95% CI: 1.67–2.84, *P*<0.05) in the low Cd group and 5.87-fold (95% CI: 5.76–6.97, *P*<0.05) in the high Cd group, compared with the control group. After 8 weeks, the kidney Cd levels had increased by 11.65-fold (95% CI: 10.37–14.84, *P*<0.05) in the low Cd group and 29.75-fold (95% CI: 20.67–34.84, *P*<0.05) in the high Cd group, compared with the control group (Figure 1D).

### Successful construction of a Cd-induced kidney damage model

**Figure 2:**
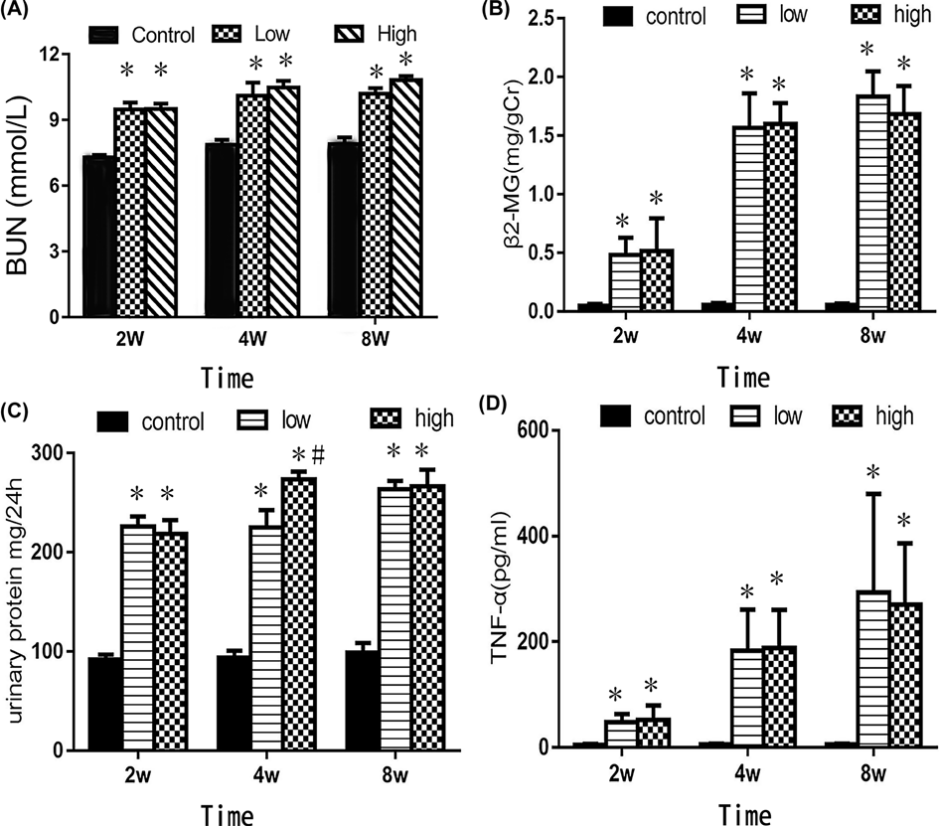
Cd-induced changes in biomarkers concentrations in the urine and blood (**A**) Effect of Cd exposure on BUN levels. (**B**) Effect of Cd exposure on urine β2-MG levels. (**C**) Effect of Cd exposure on 24 h urinary protein excretion. (**D**) Effect of Cd exposure on blood TNF-α levels. * *P*<0.05 compared with the control group. # *P*<0.05 compared with the low Cd group.

Figure 2 shows the Cd-induced changes in urine and blood biomarkers. After 2 weeks of gavage, the BUN levels were 1.31-times higher in the low Cd and high Cd groups than in the control group (95% CI: 1.21–1.4, *P*<0.05). After 4 weeks, the BUN levels of the low Cd and high Cd groups were, respectively, 1.33-fold (95% CI: 1.22–1.42, *P*<0.05) and 1.36-fold (95% CI: 1.26–1.49, *P*<0.05) higher than in the control group. Finally, after 8 weeks of gavage, the BUN levels in the low Cd and high Cd groups, respectively, reached 1.32-times (95% CI: 1.20–1.41, *P*<0.05) and 1.38-times (95% CI: 1.28–1.47, *P*<0.05) those of the control group (Figure 2A).

After 2 weeks of gavage, the urine β2-MG levels of the low Cd group were 5.44-fold higher than those of the control group (95% CI: 5.34–5.53, *P*<0.05), and those of the high Cd group were 5.67-fold higher than those of the control group (95% CI: 5.57–5.76, *P*<0.05). After 4 weeks, these levels in the low Cd and high Cd groups were, respectively, 16.1 (95% CI: 15.8–17.5, *P*<0.05) and 16.4 (95% CI: 15.2–17.5, *P*<0.05)-fold higher than in the control group. Finally, after 8 weeks of gavage, these levels reached 18.5 (low Cd group, 95% CI: 17.2–19.4, *P*<0.05) and 16.5 (high Cd group, 95% CI: 15.3–17.9, *P*<0.05) times those of the control groups (Figure 2B).

Compared with the control group, the low Cd regimen increased the 24 h urinary protein excretion by 2.51 (95% CI: 2.42–2.6, *P*<0.05), 2.44 (95% CI: 2.34–2.53, *P*<0.05), and 2.55 (95% CI: 2.45–2.64, *P*<0.05) fold after 2, 4, and 8 weeks, respectively. Meanwhile, the high Cd regimen increased them by 2.47 (95% CI: 2.36–2.57, *P*<0.05), 2.76 (95% CI: 2.64–2.84, *P*<0.05), and 2.56 (95% CI: 2.46–2.65, *P*<0.05) fold after 2, 4, and 8 weeks, respectively (Figure 2C).

Finally, we observed a similar pattern in blood TNF-α levels. Compared with the control group, the low Cd regimen increased TNF-α levels by 10.3 (95% CI: 9.5–11.2, *P*<0.05), 39.6 (95% CI: 37.5–41.8, *P*<0.05), and 56 (95% CI: 53.6–58.2, *P*<0.05) fold after 2, 4, and 8 weeks, respectively. Meanwhile, the high Cd regimen increased them by 11.5 (95% CI: 10.6–12.5, *P*<0.05), 40 (95% CI: 34.6–42.8, *P*<0.05), and 54.5 (95% CI: 51.6–55.8, *P*<0.05) fold after 2, 4, and 8 weeks, respectively (Figure 2D).

### Histopathological effects

**Figure 3:**
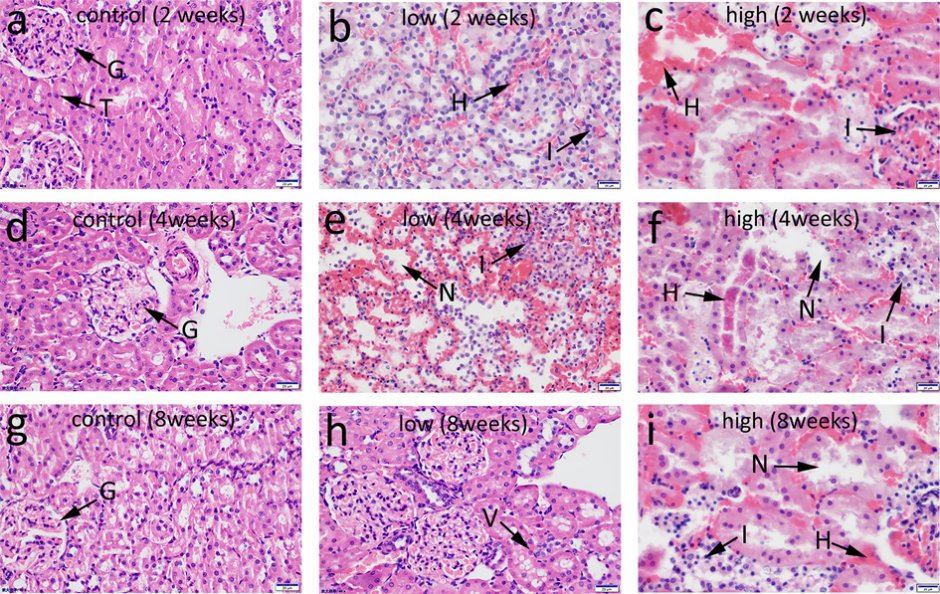
Photomicrograph of kidney sections stained with H&E for histopathological changes assessment Images of control group (**a**), low Cd group (**b**), high Cd group (**c**) at 2 weeks of intervention were obtained. Images of control group (**d**), low Cd group (**e**), high Cd group (**f**) at 4 weeks, and Images of control group (**g**), low Cd group (**h**), high Cd group (**i**) at 8 weeks. Control group images showing the normal glomeruli (G) and renal tubules (T) structures. Low Cd group images showing inflammatory cell infiltration (I), tubular cell necrosis (N), tubular vacuolization (V) (arrow), and focal hemorrhage (H). High Cd group images showing a significant reduction in tubular cell necrosis and protein cast formation (arrow) compared with the control group.

The histopathological examination of kidney tissues revealed marked changes in the Cd-treated rats. Besides, higher Cd doses and prolonged exposure resulted in more severe damage to the kidney structure (Figure 3).

Figure 3a,d,g shows the normal structure of the rat kidney observed in the control group: normal glomerular cells, no inflammatory cell infiltration, no bleeding phenomenon, and renal tubular lumen rules.

In the low Cd group, after 2 weeks, the renal tubules were swollen, and the lumen space showed inflammatory cells infiltration and some bleeding (Figure 3b). After 4 weeks, renal tubules were disordered, the lumen was destroyed, epithelial nuclei were lost, numerous cells were shed, and numerous inflammatory cells had infiltrated the lumen (Figure 3e). Finally, after 8 weeks, the lumen of renal tubules shrunk, the number of epithelial cells decreased, vacuolar degeneration was observed, and a small number of inflammatory cells infiltrated and proliferated in the glomeruli (Figure 3h).

In the high Cd group, after 2 weeks, the number of renal tubular epithelial cells decreased, epithelial cells shed, renal tubular epithelial cells showed watery degeneration, the glomerular structure blurred, and extensive bleeding and inflammatory cell infiltration occurred (Figure 3c). After 4 weeks, we observed disordered renal tubular arrangement, lumen structure disappearance, massive necrosis of renal tubular epithelial cells, visible protein tubules, obvious hemorrhage in the lumen, and inflammatory cell infiltration (Figure 3f). Finally, after 8 weeks, we observed extensive renal tubules damage and disorder, mass necrosis and disappearance of the nuclei of the epithelium of the renal tubules, extensive inflammatory cells infiltration, and watery degeneration and extensive bleeding in the epithelium of the renal tubules (Figure 3i).

### Kidney ultrastructure

**Figure 4:**
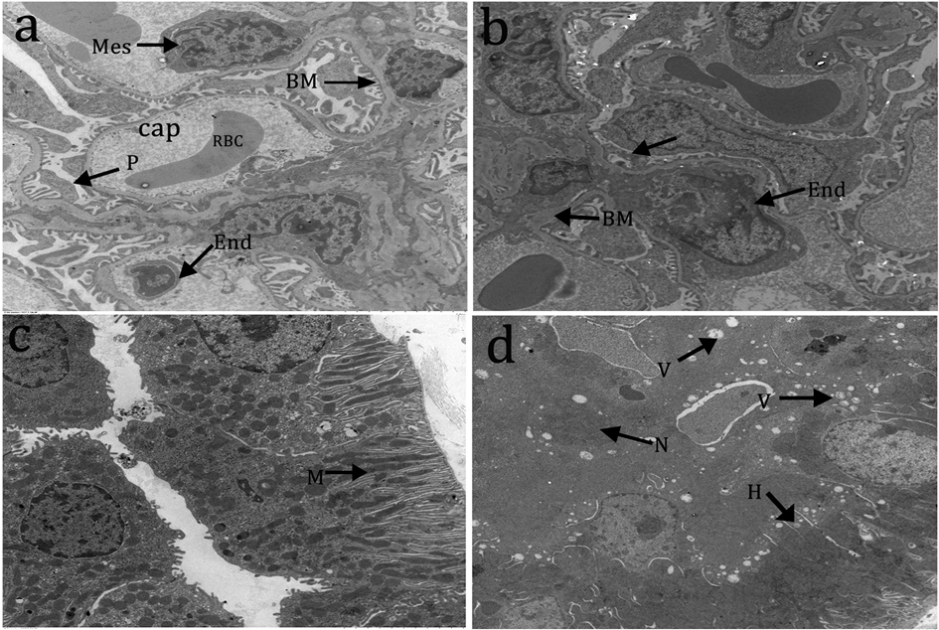
Cd-induced ultrastructural changes in renal injury TEM images of ultrathin sections (×1500). (**a,c**) Control group, showing the normal histological structure of glomeruli and renal tubules. (**b,d**) High Cd group, showing capillary endothelial cell proliferation (End), partial basement membrane thickening (BM), renal tubule lumen stenosis, mitochondrial swelling (H) in renal tubular cells, significant vacuolar degeneration (V), and lumen and coagulative necrosis (N).

We compared the ultrastructure of kidneys of rats in the control and high Cd groups after 8 weeks of treatment through TEM. We found noticeable structural changes in the glomerulus and renal tubules (Figure 4). In the control group, glomeruli had the typical Bowman capsule structure with normal capillary basement membrane, regular podocytes, capillary lumen, mesangial cell, red blood cell, and capillary endothelial cell (Figure 4a). Meanwhile, in the high Cd group, glomeruli displayed capillary endothelial cell proliferation, swelling, thickening, vascular mesangial hyperplasia, partial foot process fusion, and partial basement membrane thickening (Figure 4b). In the control group, renal tubules had a normal ultrastructure of proximal convoluted tubules, rich mitochondria, and normal, round, and regular nuclei (Figure 4c). Meanwhile, in the high Cd group, renal tubules displayed renal tubular lumen stenosis, swollen mitochondria in tubular cells, significant vacuolar degeneration, coagulation necrosis in the lumen, and swelling and disordered arrangement of microvilli on the free surface of proximal epithelial cells (Figure 4d).

### Microarray and PPI network

**Figure 5:**
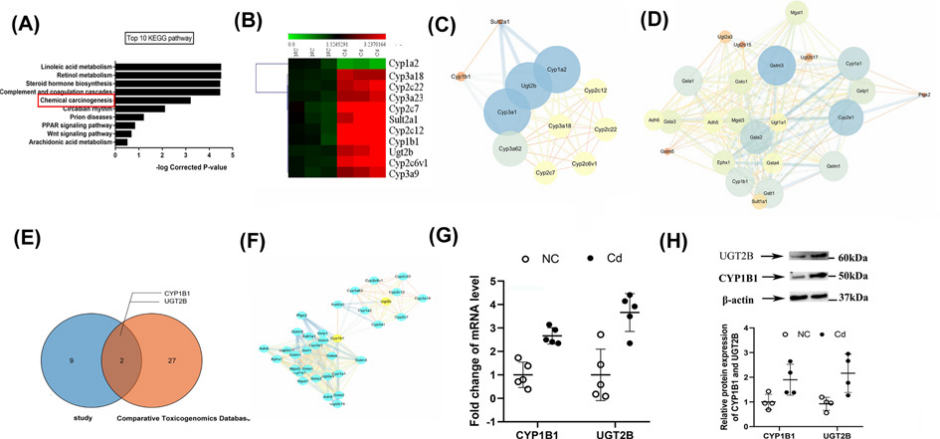
Microarray analysis and construction of PPI networks (**A**) Top ten KEGG pathways annotation. KEGG pathway enrichment. The X-axis represents the adjusted *P*-value, Y-axis is the KEGG pathway. (**B**) Heatmap of Cd-induced nephrotoxicity and the expression of related genes involved in chemical carcinogenesis pathways. Green: relatively low expression; red: relatively high expression; scale, normalized expression value. (**C**) PPI diagram of differentially expressed genes related to Cd-induced renal damage and chemical carcinogenesis pathway identified by microarray in the present study. (**D**) PPI diagram of genes related to the Cd and chemical carcinogenesis pathway in CTD. (**E**) Venn diagram showing the overlap of differentially expressed genes related to Cd-induced renal damage and chemical carcinogenesis pathway identified by microarray in the present study and the genes related to Cd-related chemical carcinogenesis pathway in the CTD database. (**F**) Merging of the PPI networks. Yellow nodes indicate the two overlapping genes. (**G**) CYP1B1 and UGT2B mRNA expression by quantitative reverse transcription polymerase chain reaction (RT-qPCR). (**H**) CYP1B1 and UGT2B expression by Western blot assay. PPI diagram, the size of a node is determined by the degree of freedom: the greater the degree of freedom, the larger the node. The color of the node is set according to the degree of freedom, going from blue (low degree of freedom) to yellow (high degree of freedom). The thickness of the edge represents the betweenness: the larger the betweenness, the thicker the edge.

Our microarray analysis revealed that 8 weeks of the high Cd regimen affected 495 mRNA transcripts in the kidney. In detail, it up-regulated 180 mRNAs and down-regulated 315 others. Using KEGG pathway analysis, we determined the pathways related to Cd-induced renal damage. The top ten paths listed by ascending *P*-value are, as shown in Figure 5A: (1) linoleic acid metabolism, (2) retinol metabolism, (3) steroid biosynthesis, (4) chemical carcinogenesis, (5) complement and coagulation cascades, (6) circadian rhythm, (7) prion diseases, (8) PPAR signaling pathway, (9) wnt signaling pathway, and (10) arachidonic acid metabolism. The genes related to the chemical carcinogenesis pathway are CYP1B1, CYP3A18, CYP2C22, CYP3A23, CYP2c7, SULT2A1, CYP2C12, CYP1A2, UGT2B, CYP2C6V1, and CYP3A9. Figure 5B represents a heat map of the role of Cd-induced renal toxicity and the expression of related genes involved in the chemical carcinogenesis pathways.

Next, we combined the STRING database with the Network Analyzer plug-in of Cytoscape to construct the PPI network of genes related to the chemical carcinogenesis pathway of Cd-induced renal toxicity shown in Figure 5C. Besides, we identified genes and chemicals involved in the Cd carcinogenesis pathway using the CTD. We found 28 related genes, analyzed the associations between proteins using STRING, and used Cytoscape to draw the PPI network shown in Figure 5D. We identified genes related to Cd-induced chemical carcinogens in CTD and drew a Venn map combining the two results. As Figure 5E shows, two overlapping genes emerged, CYP1B1 and UGT2B. Then, we merged the above two PPI networks (Figure 5F). We next quantified the expression of the two overlapping genes, CYP1B1 and UGT2B, by RT-qPCR. The results are consistent with those of the Microarray gene chip. RT-qPCR showed that the high Cd group expressed higher CYP1B1 gene (2.712-fold increase, 95% CI: 2.131–3.312) and UGT2B gene (3.665-fold increase, 95% CI: 2.412–4.662, *P*<0.05) levels than the control group (Figure 5G). Western blotting was also used to analyze and compare the protein expression differences of CYP1B1 and UGT2B in Cd-treated rat kidney tissue, as shown in Figure 5H, which was consistent with the expression results of mRNA. The present study consulted and sorted through the molecular functions of CYP1B1 and UGT2B, and lists the reports of related diseases of these two genes in previous studies and those related with nephrotoxicity, as Table 1.

**Table 1:** Molecular functions of CYP1B1 and UGT2B and reports of associated diseases

Gene	Uniprot accession	Molecular function	Related diseases	Reports related to nephrotoxicity
CYP1B1	Q64678	A cytochrome P450 monooxygenase involved in the metabolism of various endogenous substrates, including fatty acids, steroid hormones, and vitamins [30]	Chemical- and drug-induced liver injury [31]; Breast neoplasms [10]; Lung neoplasms [32]	No report? CdCl_2_ increases Cd levels [33]
UGT2B	P36511	UDP-glucuronosyltransferase (UGT) that catalyzes phase II biotransformation reactions in which lipophilic substrates are conjugated with glucuronic acid to increase the metabolite’s water solubility, thereby facilitating excretion into either the urine or bile. Essential for the elimination and detoxification of drugs, xenobiotics, and endogenous compounds [34].	Liver cirrhosis, experimental [35]; Chemical- and drug-induced liver injury [36]; Kidney diseases [37,38]	UGT2B is associated with acute renal injury [39]. However, there is no report on the role of UGT2B in Cd-induced nephropathy.

## Discussion

Cd can affect many organs, but the kidney is one of its most vulnerable targets [5]. Chronic exposure to Cd leads to gradual Cd accumulation in the epithelial cells of the proximal tubule, the main target of initial toxicity and damage [26,27]. In the present study, testing some biochemical parameters in the urine and blood revealed that the high Cd dose (6 mg/kg) was significantly nephrotoxic and increased urinary β2-MG levels and urinary protein excretion. These results are consistent with some previous studies that showed a significant increase in the concentration of biomarkers such as urinary protein excretion in Cd-exposed rats [5,15]. These biomarkers are observed in the urine after proximal tubular injury, and reabsorption dysfunction usually indicates tubular epithelial injury [5,15,28,29]. We also observed that levels of nephrotoxicity indicators, such as BUN and TNF-α, were higher in the Cd-exposed groups than in the control group. This indicates that the rat gavage yielded a successful Cd nephrotoxicity animal model.

Meanwhile, the histopathological examination showed that Cd administration caused progressive tubular and glomerular necrosis, which is consistent with the Cd-induced histopathological changes in the kidney reported by Aqeel et al. [2,5,16]. The renal tubular changes induced by Cd poisoning may be associated with hydraulic changes in renal tissue [14].

Cd poisoning can partially alter intracellular ion transport in the renal tubules, resulting in extensive pathological changes, including nuclei loss, massive necrosis of tubular epithelial cells, inflammatory cell infiltration, and hydropic degeneration of the tubular epithelium. Then, we explored the Cd-induced ultrastructure renal damage by TEM. The images revealed numerous abnormalities in the glomeruli and tubules of the Cd-treated kidneys compared with the control group. The ultrastructure of the rat kidney tissue in the Cd gavage groups was significantly altered. For example, the glomerular thylakoid proliferated, some pedicles fused, and some basement membranes thickened. In addition, the tubular lumen narrowed, and the mitochondria within the tubular cells swelled. Besides, we noted marked vacuolar degeneration, intratubular coagulation necrosis, proximal epithelial cell-free surface vascular interstitial hyperplasia, and microvilli swelling and disorganization. The results of the microarray indicated that genes from kidney tissue in this model could be enriched in the chemical carcinogenic pathway, when no significant kidney tissue carcinogenesis was observed morphologically. We therefore believe that these early genes, which enriched in the chemical carcinogenic pathway, may be the early biomarkers of Cd-induced precancerous lesions in the kidney.

We further analyzed the differential mRNAs in this pathway by PPI network analysis and compared with the CTD database of proteins associated with the Cd-induced chemical carcinogenesis pathway. We found two overlapping genes (CYP1B1 and UGT2B). Cd up-regulates CYP1B1 and inhibits CYP1A2. CYP1B1 encodes a member of the cytochrome P450 superfamily of enzymes [30]. CYP1B1 is mainly expressed in extrahepatic organs and has a pivotal role in the metabolic activation of carcinogens [30,31]. In cancer cells and the cancer microenvironment, CYP1B1 promotes cancer development by activating xenobiotics and steroids, producing proinflammatory and angiogenic factors, and directly regulating endothelial cell angiogenesis [32]. Combined with previous studies, the morphological (from the histological and TEM experiments) findings on kidney injury of the present study suggest that CYP1B1 plays a key role in cancer angiogenesis, acting both in cancer cells and the tumor microenvironment [32].

Glucuronidation by UGT2Bs is a major pathway for the elimination of endobiotics and xenobiotics, including therapeutic drugs [33]. Meanwhile, previous studies have linked UGT2B to kidney diseases, and some compounds even alleviate kidney diseases through UGT2B [34,35]. UGT2B has been reported to be associated with acute renal injury [36].

Taken together, these two genes may be potential biomarkers prior to Cd-induced renal carcinogenesis.

One limitation of the present study is the absence of *in vivo* experiments to explore the function of these two genes in Cd-induced renal carcinogenesis. However, we established a Cd-induced kidney injury rat model that allowed us to document the pathomorphological and ultrastructural alterations of Cd-induced kidney injury. We also conducted a preliminary exploration of the pathways of precancerous lesions and key biomarkers of Cd-induced kidney injury using gene chip technology combined with the CTD database.

## Data Availability

David (https://david.ncifcrf.gov/Summary). STRING (http://string-db.org). CTD (http://ctdbase.org/). All data generated or analyzed during the present study were included in the present published article.

## Abbreviations

β2-MG: β2-microglobulin
BM: basement membrane thickening
BUN: blood urea nitrogen
Cd: cadmium
CdCl2: Cadmium chloride
CTD: Comparative Toxicogenomics Database
dsDNA: double-stranded complementary DNA
GFR: glomerular filtration rate
H&E: hematoxylin and eosin
PPI: protein–protein interaction
RT-qPCR: quantitative reverse transcription polymerase chain reaction
TEM: transmission electron microscopy
TNF-α: tumor necrosis factor-α
UGT: UDP-glucuronosyltransferase
UGT2B: UDP-glucuronyltransferase 2B
